# Supplementary material for: Temperature-driven coordination of circadian transcriptome regulation
Source: bioRxiv. 2023 Nov 1:2023.10.27.563979. Preprint. [Version 1] doi: 10.1101/2023.10.27.563979 (PMC10634908; doi:10.1101/2023.10.27.563979)
Supplement: Supplement 1 [file NIHPP2023.10.27.563979v1-supplement-1.pdf]

bioRxiv preprint doi: https://doi.org/10.1101/2023.10.27.563979; this version posted November 1, 2023. The copyright holder for this preprint (which was not certified by peer review) is the author/funder, who has granted bioRxiv a license to display the preprint in perpetuity. It is made available under aCC-BY 4.0 International license.

8 Supplemental material

Comparison against JTK-CYCLE

JTK-CYCLE was used to validate the performance of harmonic regression. For the V2 experiment, replicates were averaged first before the standard JTK-CYCLE protocol with period 24 hr and sampling interval 2 hrs. p values computed from JTK-CYCLE were highly correlated with those computed using harmonic regression (log linear regression: V1 25°C slope: 0.81; V1 18°C slope: 0.85; V2 25°C slope: 0.69; V2 18°C slope: 0.80; supplemental Figure S1B).

Read alignment

Basic quality checking of sequence files was performed with FastQC [27]. Paired-end reads were first trimmed using Atropos version 1.1.31 [28] using the options

```
atropos trim --aligner insert -a AGATCGGAAGAGCACACGTCTGAACTCCAGTCA \
       -A AGATCGGAAGAGCGTCGTGTAGGGAAAGAGTGT --minimum-length 50
```

Reads were then aligned and quantified using STAR [29] (version STAR_2.7.10a_alpha_220818) and RSEM [30] (version 1.3.1). STAR and RSEM indexes were first built using the Ensembl *Drosophila melanogaster* BDGP6.32 reference (release 107) using standard parameters. STAR was used with the options

```
--outFilterType BySJout --alignIntronMax 1000000 \
--quantMode GeneCounts TranscriptomeSAM
```

to produce raw counts and also a BAM file with reads aligned to transcriptome. RSEM was then used with options `--paired-end --strandedness none` to produce tags-per-million (TPM) counts for each gene from transcriptome alignments. Postprocessing of the count data into table form was performed with custom Perl, Python and shell

bioRxiv preprint doi: https://doi.org/10.1101/2023.10.27.563979; this version posted November 1, 2023. The copyright holder for this preprint (which was not certified by peer review) is the author/funder, who has granted bioRxiv a license to display the preprint in perpetuity. It is made available under aCC-BY 4.0 International license.

scripts.

Because FastQC reported significant sequence duplication in the samples, we also performed the same analysis as above after deduplicating the reads. First, a BAM file was created using STAR with the options

```
--outFilterType BySJout --alignIntronMax 1000000 \
--outSAMmultNmax 1 --outSAMtype BAM SortedByCoordinate
```

to produce only uniquely mapped reads, and then duplicate paired-end reads were removed using bamUtil [32] with the options

```
bam dedup --rmDups --excludeFlags 0xB04 --oneChrom
```

The resulting reads were then re-aligned with STAR to produce a BAM file with reads aligned to the transcriptome, followed by RSEM with the same options as above to produce TPM counts. De-duplication significantly decreased the number of reads with large TPM values (e.g., > 100), and as the result the TPM values of genes with smaller TPM values were increased by approximately a factor of 1.6-1.7. Deduplicated reads were used throughout the paper.

Single-end reads were quality-assessed with FastQC and then trimmed with Atropos using the options

```
atropos -a AGATCGGAAGAGCACACGTCTGAACTCCAGTCAC --minimum-length 16
```

Reads were then aligned and quantified using STAR with the same options as before,

```
--outFilterType BySJout --alignIntronMax 1000000 \
--quantMode GeneCounts TranscriptomeSAM
```

bioRxiv preprint doi: https://doi.org/10.1101/2023.10.27.563979; this version posted November 1, 2023. The copyright holder for this preprint (which was not certified by peer review) is the author/funder, who has granted bioRxiv a license to display the preprint in perpetuity. It is made available under aCC-BY 4.0 International license.

followed by RSEM with the options `--strandedness none`.

bioRxiv preprint doi: https://doi.org/10.1101/2023.10.27.563979; this version posted November 1, 2023. The copyright holder for this preprint (which was not certified by peer review) is the author/funder, who has granted bioRxiv a license to display the preprint in perpetuity. It is made available under aCC-BY 4.0 International license.

556 ## 8.1 Figure S1

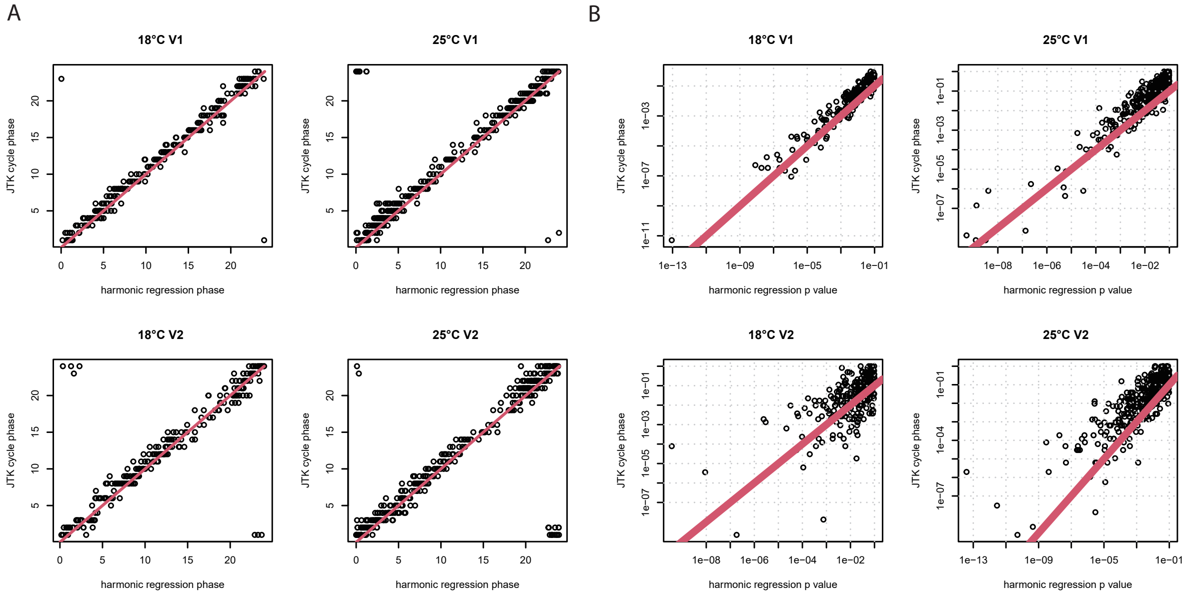

Figure S1: Comparison of harmonic regression and JTK-cycle. A: Phases of cycling genes estimated using harmonic regression and JTK-CYCLE. B: p values estimated using harmonic regression and JTK-CYCLE.

bioRxiv preprint doi: https://doi.org/10.1101/2023.10.27.563979; this version posted November 1, 2023. The copyright holder for this preprint (which was not certified by peer review) is the author/funder, who has granted bioRxiv a license to display the preprint in perpetuity. It is made available under aCC-BY 4.0 International license.

8.2 Figure S2

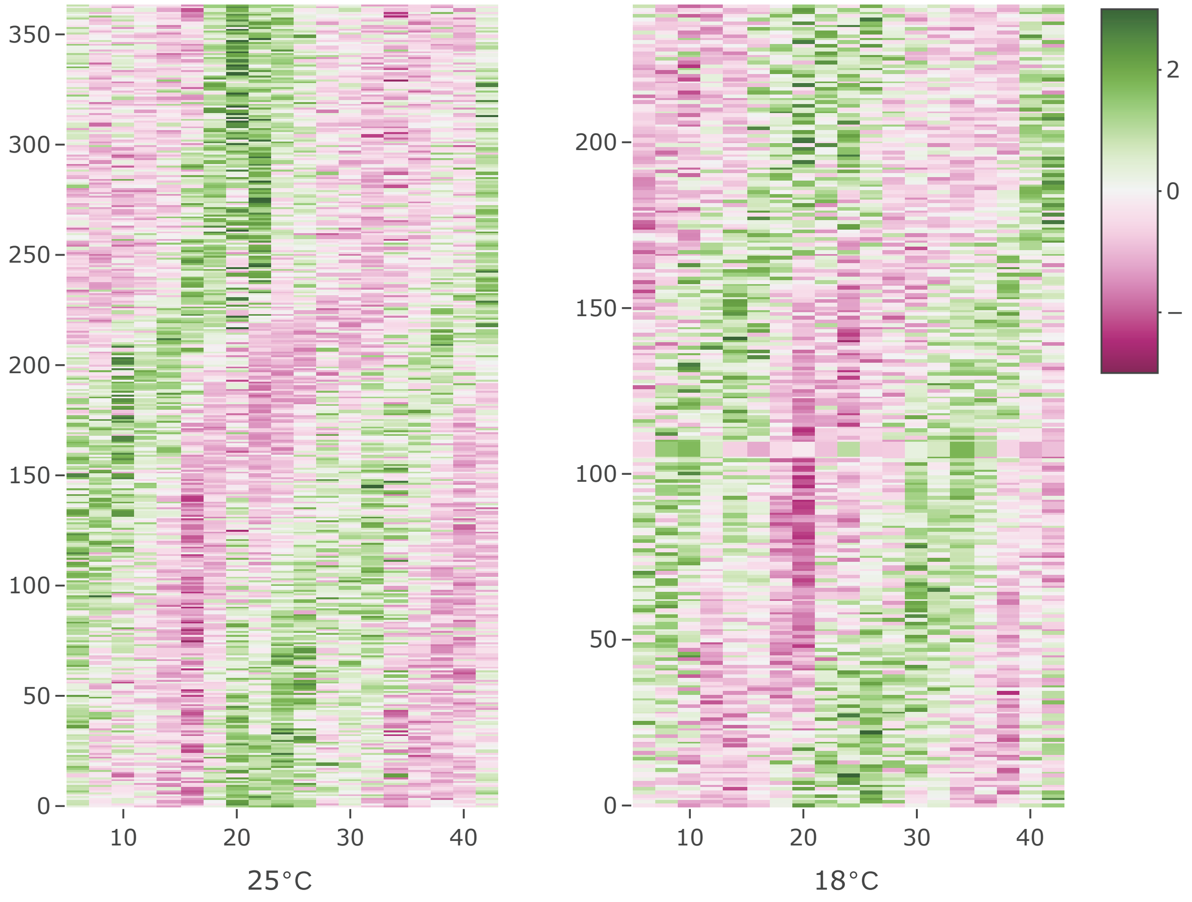

Figure S2: Heatmap showing the Z-scored TPM of the identified cycling genes in the V2 experiment. For visualization purposes, replicates were averaged.

bioRxiv preprint doi: https://doi.org/10.1101/2023.10.27.563979; this version posted November 1, 2023. The copyright holder for this preprint (which was not certified by peer review) is the author/funder, who has granted bioRxiv a license to display the preprint in perpetuity. It is made available under aCC-BY 4.0 International license.

558 ## 8.3 Figure S3

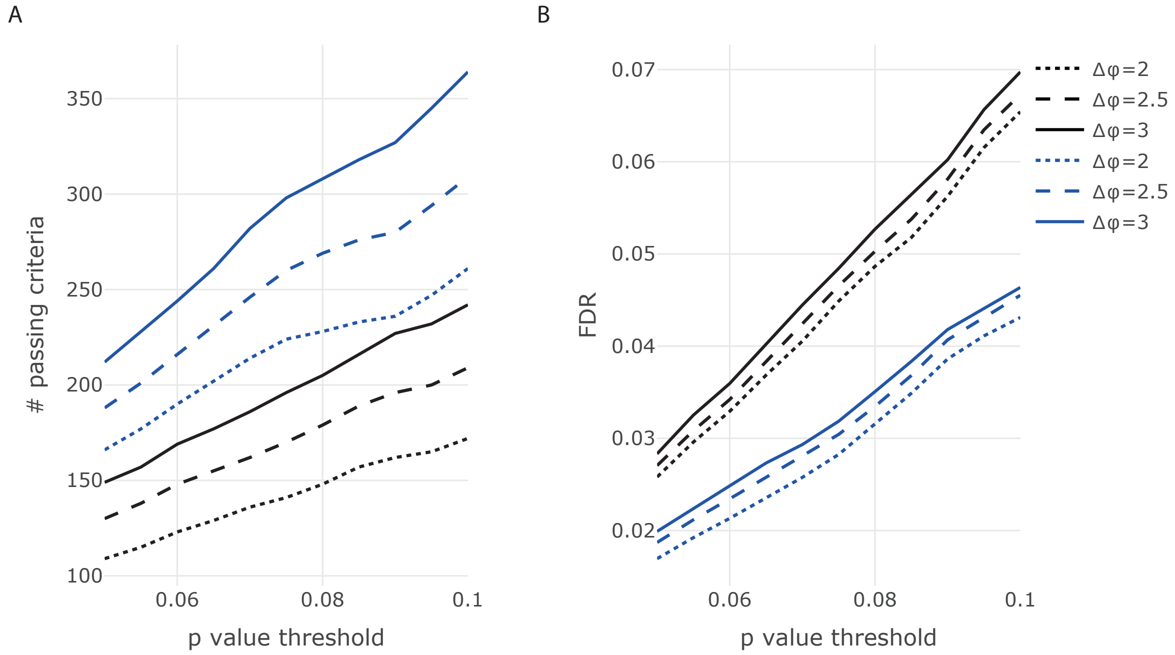

Figure S3: Number of identified cycling genes (A) and false discovery rate (B) as a function of harmonic regression p-value thresholds under different $\Delta\phi$ thresholds. Blue: 25°C. Black: 18°C. Our selected thresholds, $p < 0.1$ and $\Delta\phi < 3$ yields FDRs of 0.07 and 0.047 in 18°C and 25°C, respectively.

bioRxiv preprint doi: https://doi.org/10.1101/2023.10.27.563979; this version posted November 1, 2023. The copyright holder for this preprint (which was not certified by peer review) is the author/funder, who has granted bioRxiv a license to display the preprint in perpetuity. It is made available under aCC-BY 4.0 International license.

559

8.4 Figure S4

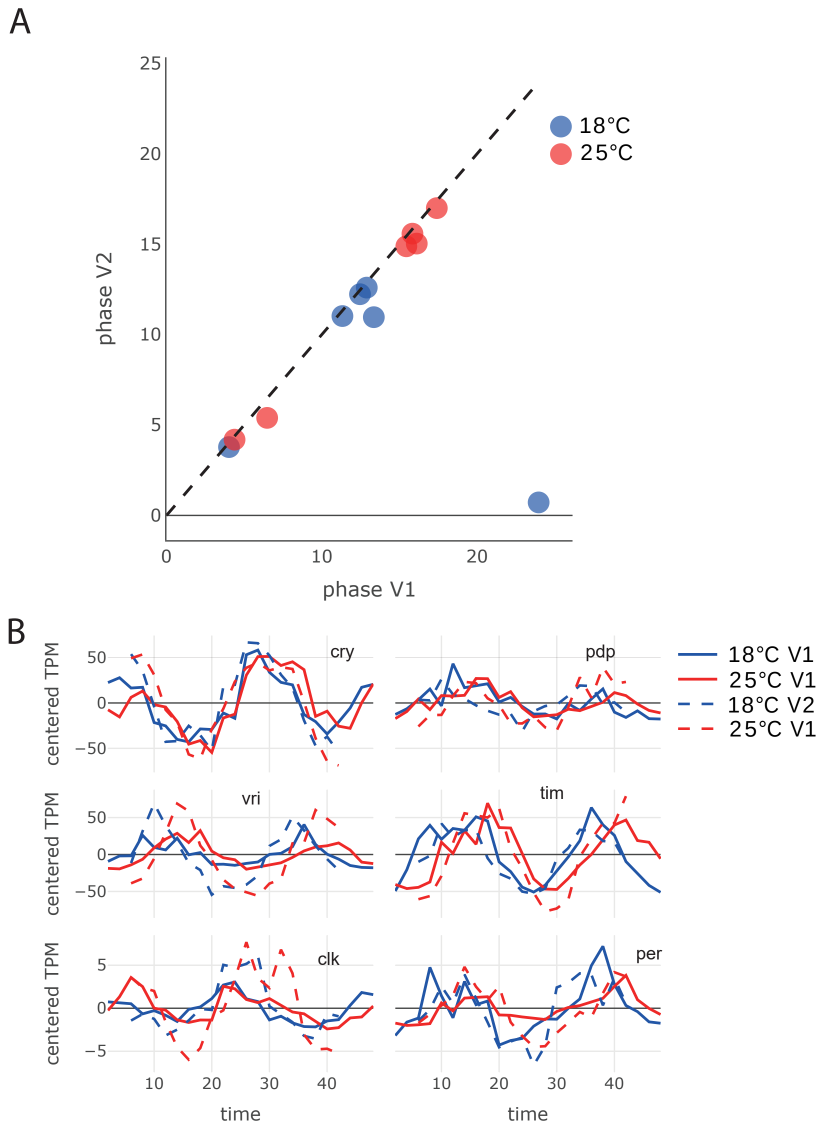

Figure S4: A: Phases of the core clock genes estimated in the V1 and V2 experiments, dashed line indicates $y = x$. C: Centered TPM of core clock genes. Replicates were concatenated for V1 and averaged for V2 for better visualization.

bioRxiv preprint doi: https://doi.org/10.1101/2023.10.27.563979; this version posted November 1, 2023. The copyright holder for this preprint (which was not certified by peer review) is the author/funder, who has granted bioRxiv a license to display the preprint in perpetuity. It is made available under aCC-BY 4.0 International license.

560 ## 8.5 Figure S5

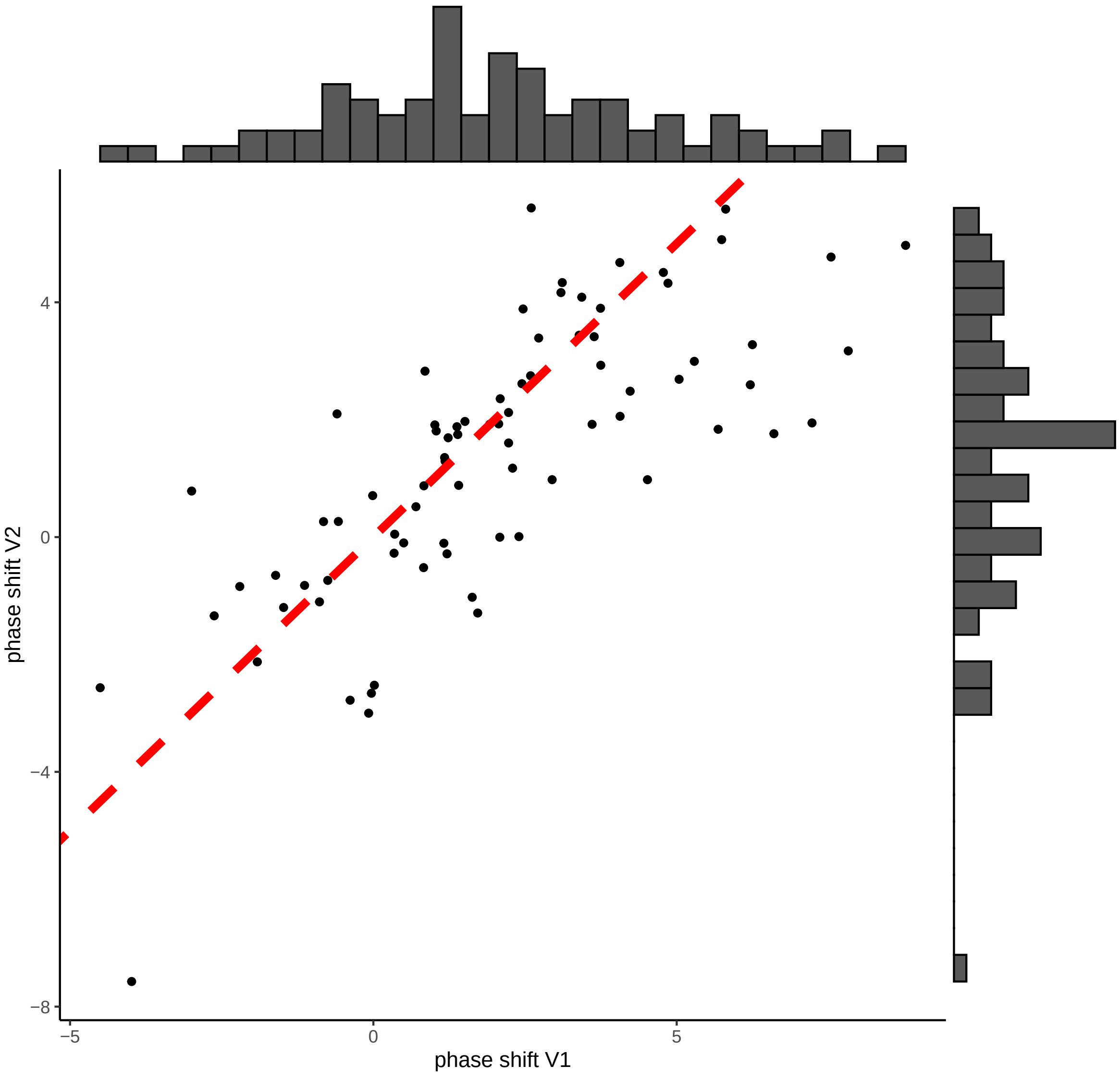

Figure S5: Estimated phase shift of genes that cycle under both temperatures (estimated via limorhyde2). Red line indicates $y = x$

bioRxiv preprint doi: https://doi.org/10.1101/2023.10.27.563979; this version posted November 1, 2023. The copyright holder for this preprint (which was not certified by peer review) is the author/funder, who has granted bioRxiv a license to display the preprint in perpetuity. It is made available under aCC-BY 4.0 International license.

561 ## 8.6 Figure S6

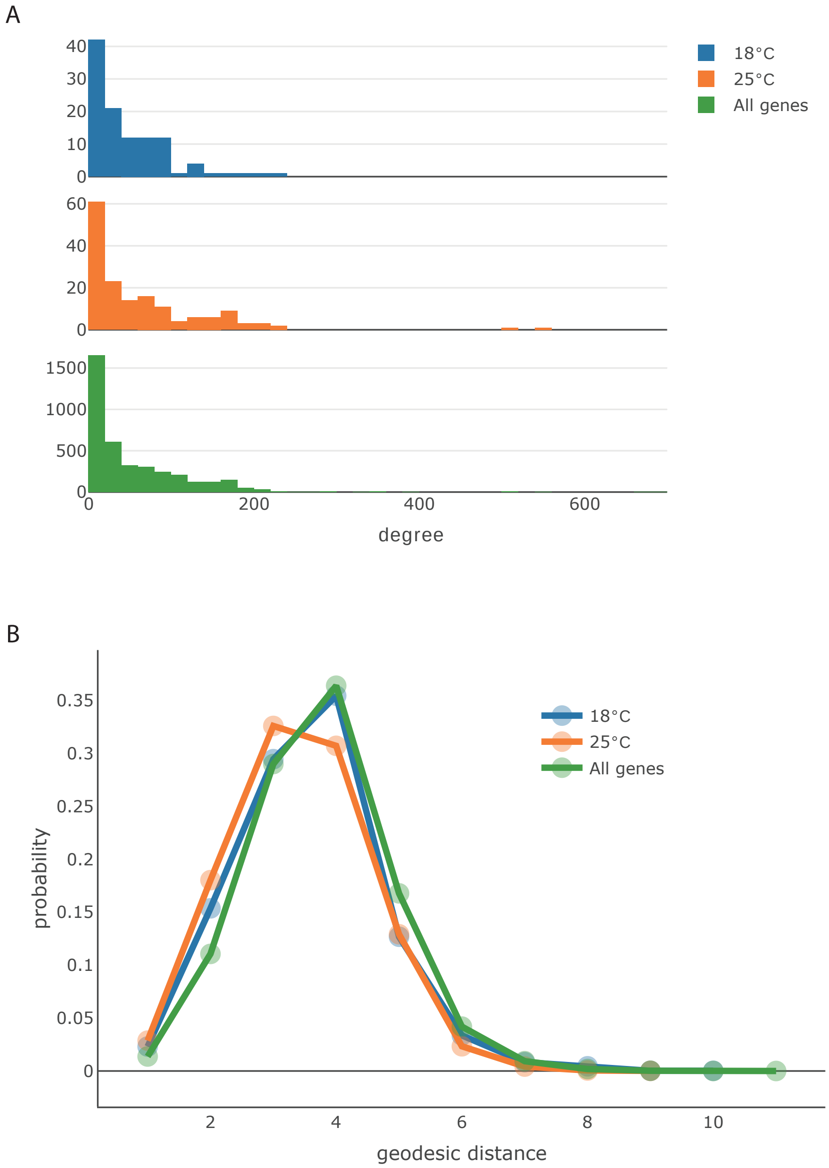

Figure S6: Network properties of cycling genes. A: Degree distributions of genes detected as cycling under the two temperatures, as well as all genes. B: Geodesic (network) distance distributions for gene pairs that are cycling under the two temperatures, as well as all gene pairs.

bioRxiv preprint doi: https://doi.org/10.1101/2023.10.27.563979; this version posted November 1, 2023. The copyright holder for this preprint (which was not certified by peer review) is the author/funder, who has granted bioRxiv a license to display the preprint in perpetuity. It is made available under aCC-BY 4.0 International license.

562 ## 8.7 Figure S7

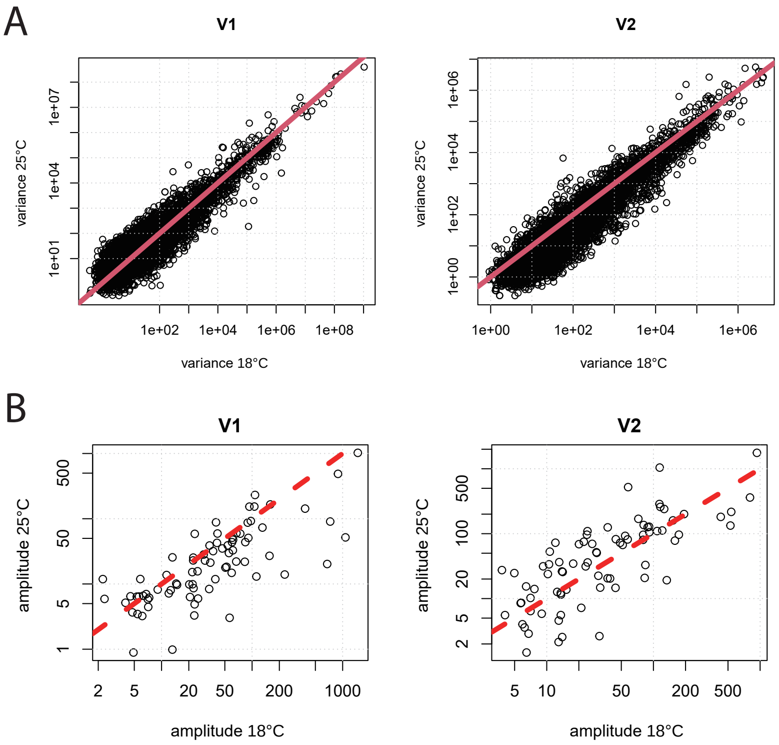

Figure S7: Comparison of variance and amplitude in 25°C relative to 18°C in the two datasets. A: Gene expression variance for all genes passing filtration in V1 and V2. B: Oscillation amplitude of genes cycling under both temperatures in V1 and V2. In all plots, red lines indicate $y = x$.
